# Pinitol Improves Lipopolysaccharide-Induced Cellular Damage in Human Dermal Microvascular Endothelial Cells

**DOI:** 10.3390/molecules30071513

**Published:** 2025-03-28

**Authors:** Min Young Go, Jinsick Kim, Chae Young Jeon, Mujun Kim, Dong Wook Shin

**Affiliations:** Research Institute for Biomedical and Health Science, Konkuk University, Chungju 27478, Republic of Korea; rhalsdud1011@kku.ac.kr (M.Y.G.); jindoli477@kku.ac.kr (J.K.); young4man@kku.ac.kr (C.Y.J.); besy100@kku.ac.kr (M.K.)

**Keywords:** human dermal microvascular endothelial, pinitol, antioxidant, anti-inflammation, lipopolysaccharide, endothelial function

## Abstract

3-O-Methyl-D-chiro-inositol (pinitol) has been reported to possess insulin-like effects and is known as one of the anti-diabetic agents for improving muscle and liver function. However, the beneficial effects of pinitol on human dermal microvascular endothelial cells (HDMECs) are not well understood. In this study, we investigated whether pinitol could protect HDMECs from damage induced by lipopolysaccharides (LPSs), which cause various cell defects. We observed that pinitol enhanced wound healing for LPS-damaged HDMECs. We found that pinitol significantly downregulated the LPS-induced upregulation of reactive oxygen species (ROS). Pinitol also significantly restored the mitochondrial membrane potential in these cells. Immunofluorescence analysis revealed that pinitol notably reduced the nuclear localization of NF-κB in LPS-damaged HDMECs. Furthermore, we demonstrated that pinitol decreased the phosphorylation levels of the MAPK family in LPS-damaged HDMECs. Interestingly, we observed that pinitol improved tube formation in LPS-damaged HDMECs. Taken together, we suggest that pinitol exerts several beneficial effects on LPS-damaged HDMECs and may be a promising therapeutic agent for improving vascular-related skin diseases.

## 1. Introduction

Blood vessels are important pathways that deliver oxygen and nutrients to all organs and tissues of the body [1,2]. They play a crucial role during the growth stage, regeneration, and wound healing [3]. After tissue damage, blood vessels begin the healing process by delivering growth factors and blood coagulation factors such as fibrin to the wound [4,5]. This process triggers the formation of new blood vessels from the existing vascular network [6,7]. The newly formed blood vessels continue to supply blood to the regenerated tissues, thereby restoring the structure and function of the tissues and organs [8].

Vascular diseases can arise due to inflammation, damage, and disturbances in blood flow, which are often accompanied by various types of skin diseases and lesions [9,10,11,12] such as psoriasis [13], primary Raynaud’s disease [14], and scleroderma [15]. Diabetic foot ulcers (DFUs) are also one of the serious vascular complications of diabetes [16]. A high blood glucose environment triggers the release of ROS and inflammatory cytokines, leading to vascular damage and chronic inflammation, which results in ulcer formation [17].

Human dermal microvascular endothelial cells (HDMECs) are the major endothelial cells of human skin, surrounding dermal capillaries and microvessels [18,19]. The physiological function of endothelial cells is to maintain a healthy vascular system by controlling vascular permeability, inflammation, and angiogenesis [20,21]. The endothelial cells around dermal capillaries become activated in response to skin damage or inflammation, and various cytokines induce cell movement to the damaged area [22,23]. HDMECs play a crucial role in skin regeneration and inflammatory responses [24]. When HDMECs are damaged by internal or external causes, vascular-related skin diseases can occur. Thus, there is a need to discover therapeutic agents that can restore their function.

Pinitol is a type of sugar alcohol derived from leguminous plants [25]. This active phytochemical is considered an ideal compound due to its non-toxic nature [26]. Pinitol is used as an anti-diabetic agent because it is involved in the insulin signaling pathway [27,28]. In addition, its efficacy as an antioxidant, anti-inflammatory, and anti-cancer agent has been reported [29,30]. In our previous experiments, we found that pinitol mitigates damage in fibroblasts caused by ultraviolet radiation [31], and we demonstrated that it ameliorates wound healing in DFU animal models [32]. However, the effects of pinitol on endothelial function in LPS-induced vascular damage remain unknown.

In this study, we investigate whether pinitol improves endothelial function in HDMECs exposed to LPS-induced inflammation and oxidative stress.

## 2. Results

### 2.1. Effect of Pinitol on the Cell Viability in HDMECs

**Figure 1:**
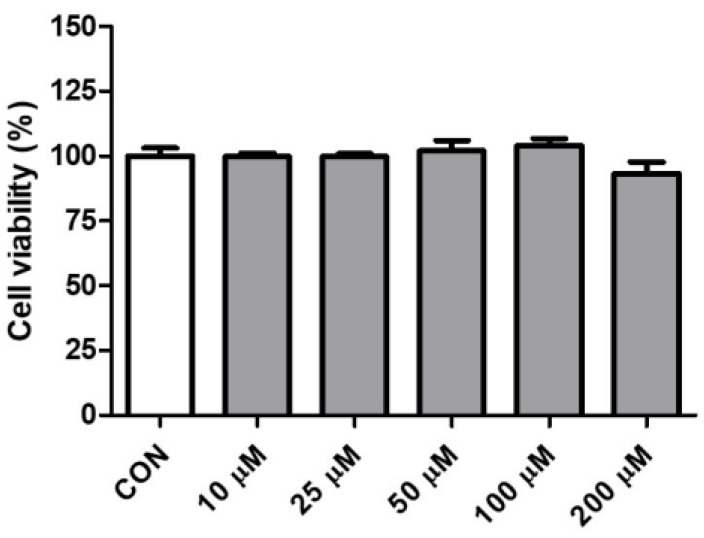
Cell viability of pinitol in HDMECs. The cell viability of pinitol in HDMECs was measured with an EZ-cytox assay. The pinitol was treated for 24 h and had more than 100% cell viability at concentrations of 10, 25, 50, and 100 μM. Cell viability was calculated as a percentage (%) compared with controls. All data are expressed as means ± SD (*n* = 3).

An EZ-cytox assay was performed to find the appropriate concentration that did not show cytotoxicity. Pinitol was applied to HDMECs at various concentrations (10, 25, 50, 100, and 200 μM) for 24 h. The results showed that pinitol demonstrated over 100% cell viability in HDMECs up to a concentration of 100 μM (Figure 1). Consequently, concentrations of 10, 50, and 100 μM were selected.

### 2.2. Pinitol Improved Wound Healing in LPS-Damaged HDMECs

Wound healing involves the hemostasis and inflammation phase, the proliferative phase, and the remodeling phase [33]. One key aspect of wound healing is the supply of nutrients, immune cells, and oxygen through angiogenesis [34]. Wound healing abnormalities, such as excessive healing or chronic wounds, can impair normal body functions [35]. LPS, a pro-inflammatory stimulus, is commonly used to simulate both systemic and localized infections in various types of tissues [36,37]. LPS can weaken vascular integrity in endothelial cells [38]. We treated pinitol, an anti-diabetic substance, to evaluate its effects on blood vessel improvement and utilized metformin as a positive control.

**Figure 2:**
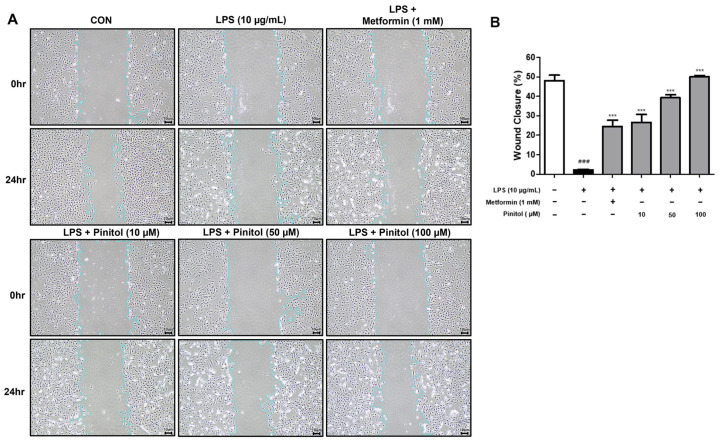
Pinitol enhanced wound healing in LPS-damaged HDMECs. The wound healing assay was conducted with LPS-damaged HDMECs. (**A**) The concentration-dependent wound-healing effects of pinitol (10, 50, and 100 μM) and (**B**) wound closure (%) were measured by the rate of cell migration into the scratched area over time using ImageJ™ software version 2.9.0. All data are expressed as mean ± SD (*n* = 3). Scale bar = 10 µm. *** *p* < 0.001 compared with only LPS-damaged group. ### *p* < 0.001 compared with the control group.

Therefore, we conducted a wound-healing assay to assess the wound-healing effect of pinitol in HDMECs. HDMECs were scratched and treated with a concentration of LPS (10 μg/mL), metformin, and pinitol, respectively. After 24 h, the LPS-damaged group showed no significant difference in the degree of wound closure. In contrast, treatment with pinitol significantly increased wound closure (Figure 2). These results suggest that pinitol significantly enhanced the wound-healing effect in LPS-damaged HDMECs in a concentration-dependent manner.

### 2.3. Pinitol Recovered ROS Levels in LPS-Damaged HDMECs

**Figure 3:**
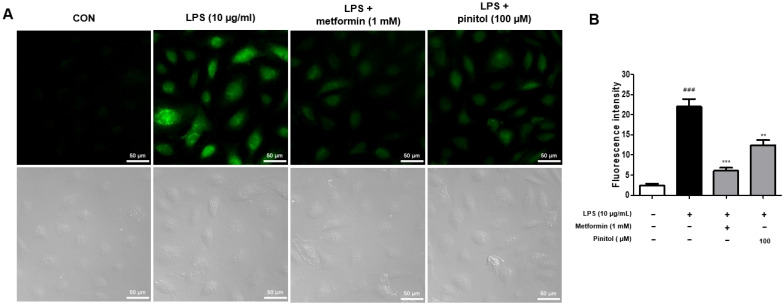
Pinitol decreased ROS levels in LPS-damaged HDMECs. The DCF-DA assay was conducted with LPS-damaged HDMECs. (**A**) LPS (10 μg/mL) and metformin (1 mM) or pinitol (100 μM) were co-treated for 24 h. These images show the ROS-reducing effect of pinitol. Scale bar = 50 µm. (**B**) Fluorescence intensity was measured using ImageJ™ software version 2.9.0. All data are expressed as mean ± SD (*n* = 3). ** *p* < 0.01 compared with the LPS-damaged group. *** *p* < 0.001 compared with the LPS-damaged group. ### *p* < 0.001 compared with the control group.

Oxidative stress refers to the excessive production of ROS in the body due to various harmful stimuli. If high levels of ROS persist for a prolonged period, then imbalances in the oxidative and antioxidant systems can lead to vascular dysfunction, such as endothelial dysfunction, promotion of inflammatory reactions, or vascular complications [39,40]. We evaluated the potential of pinitol to inhibit the ROS production induced by LPS in HDMECs. As expected, LPS increased the ROS levels. However, treatment with pinitol significantly downregulated the intracellular ROS levels in LPS-damaged HDMECs (Figure 3).

### 2.4. Pinitol Ameliorated the Membrane Potential of Mitochondria in LPS-Damaged HDMECs

**Figure 4:**
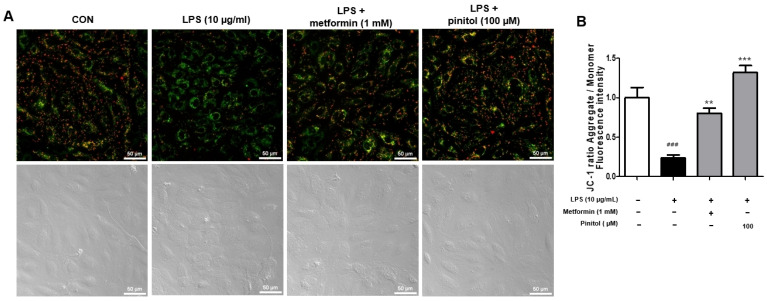
Pinitol restored the membrane potential of mitochondria in the LPS-damaged HDMECs. The JC-1 assay was conducted with LPS-damaged HDMECs. (**A**) LPS (10 μg/mL) and metformin (1 mM) or pinitol (100 μM) were co-treated for 24 h. Each image of JC-1 was captured using a Nikon Eclipse Ti2 fluorescence live-cell imaging microscope. Scale bar = 50 µm. (**B**) J-monomers (green) and J-aggregates (red) fluorescence intensity were quantified using ImageJ™ software version 2.9.0. All data were expressed as mean ± SD (*n* = 3). ** *p* < 0.01 compared with only the LPS-damaged group. *** *p* < 0.001 compared with only the LPS-damaged group. ### *p* < 0.001 compared with the control group.

Mitochondria are key organelles for cellular energy production, and the mitochondrial membrane potential serves as an important indicator of cell health and functional status [41,42]. LPS induces ROS production, leading to a decrease in the mitochondrial membrane potential [43,44]. We assessed the mitochondrial membrane potential to determine whether pinitol is associated with mitochondrial health and functional status. The stronger the red fluorescence, the more actively the mitochondria are functioning, indicating a healthy cell state. An increase in green fluorescence suggests that mitochondrial function has declined due to cell stress or damage [45]. The degree of mitochondrial depolarization was assessed by the ratio of red to green fluorescence intensity [46]. As expected, LPS caused an increase in green fluorescence compared with the control group. In contrast, treatment with pinitol led to an increase in red fluorescence, indicating that pinitol restored the mitochondrial potential in LPS-damaged HDMECs (Figure 4).

### 2.5. Pinitol Inhibited NF-κB Pathway in LPS-Damaged HDMECs

**Figure 5:**
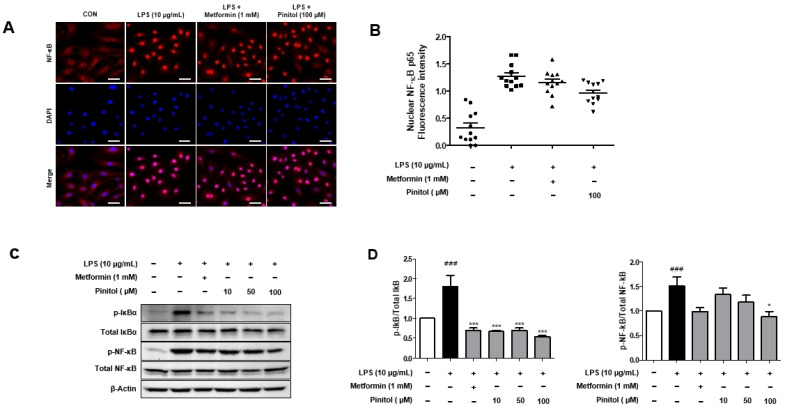
Pinitol downregulated the phosphorylation level of IκB and NF-kB in LPS-damaged HDMECs. Immunofluorescence was conducted on LPS-damaged HDMECs. (**A**) LPS (10 μg/mL), metformin (1 mM), or pinitol (10, 50, and 100 μM) was applied for 24 h. Each immunofluorescence image was captured using a Nikon Eclipse Ti2 fluorescence live-cell imaging microscope. Scale bar = 50 µm. (**B**) The percentage of NF-kB-positive nucleic cells was quantified by using ImageJ version 2.9.0. (**C**) Western blot analysis of the NF-kB pathway, measuring the IκB and NF-kB protein expression levels in LPS-damaged HDMECs. (**D**) Each protein level was normalized to actin expression. All data are expressed as mean ± SD (*n* = 3). * *p* < 0.05 compared with the LPS-damaged group. *** *p* < 0.001 compared with the LPS-damaged group. ### *p* < 0.001 compared with the control group.

LPS binds to TLR4 receptors on the cell surface, regulating the IκB/NF-κB signaling pathway [47]. NF-κB is present in the cytoplasm, being bound to the inhibitory protein IκB. Activation of IκB kinases leads to the phosphorylation of IκB, causing its dissociation from NF-κB and the translocation of its subunits into the nucleus [48,49]. This increases inflammation and can activate oxidative stress and inflammatory cascades [50]. We investigated whether pinitol could inhibit the NF-κB pathway. Immunofluorescence staining revealed that LPS resulted in the translocation of NF-kB into the nucleus in HDMECs. In contrast, pinitol decreased the translocation of NF-kB (Figure 5A,B). LPS significantly increased the phosphorylation levels of IκB and NF-κB. However, treatment with pinitol reversed this effect by downregulating the protein phosphorylation levels of p-IκB-α and p-NF-κB (Figure 5C,D). These data suggest that pinitol ameliorated the inflammatory response by inhibiting the NF-κB pathway.

### 2.6. Pinitol Significantly Decreased the Phosphorylation Levels of MAPKs in LPS-Damaged HDMECs

**Figure 6:**
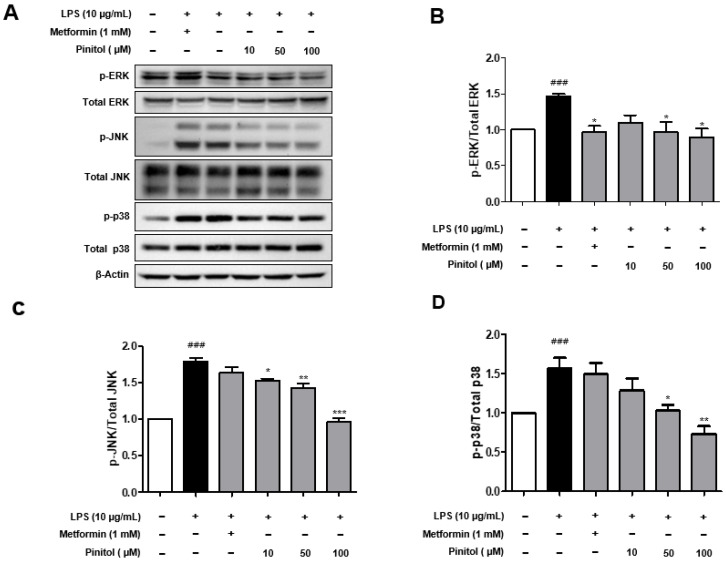
Pinitol inhibited phosphorylation of the MAPK pathway in LPS-damaged HDMECs. (**A**) Western blotting analysis of the MAPKs was conducted on LPS-damaged HDMECs. LPS (10 μg/mL) and metformin (1 mM) or pinitol (10, 50, and 100 μM) were co-treated for 24 h. Phosphorylation or dephosphorylation status of (**B**) ERK, (**C**) JNK, and (**D**) p38 in LPS-damaged HDMECs. Each relative protein level was normalized to actin expression. All data are expressed as mean ± SD (*n* = 3). * *p* < 0.05 compared with the LPS-damaged group. ** *p* < 0.01 compared with the LPS-damaged group. *** *p* < 0.001 compared with the LPS-damaged group. ### *p* < 0.001 compared with the control group.

In addition to the NF-κB pathway, the mitogen-activated protein kinase (MAPK) pathway is an important regulator of cell growth, survival, and inflammatory responses [51]. Previous studies have reported that inhibition of the MAPK pathway reduces the activity of IKK, a key upstream protein in the NF-κB signaling pathway, ultimately blocking the amplification of endothelial inflammation [52,53,54]. We examined whether pinitol inhibited the activity of MAPKs in LPS-damaged HDMECs. The phosphorylation levels of MAPKs, including ERK, p38, and JNK, increased in LPS-damaged HDMECs (Figure 6). In contrast, treatment with pinitol resulted in decreased phosphorylation of ERK, p38, and JNK in LPS-damaged HDMECs (Figure 6). These results suggest that pinitol effectively inhibited the phosphorylation of MAPKs in the LPS-damaged group, implicating this pathway as a key regulator of vascular inflammation.

### 2.7. Pinitol Improved Tube Formation in LPS-Damaged HDMECs

**Figure 7:**
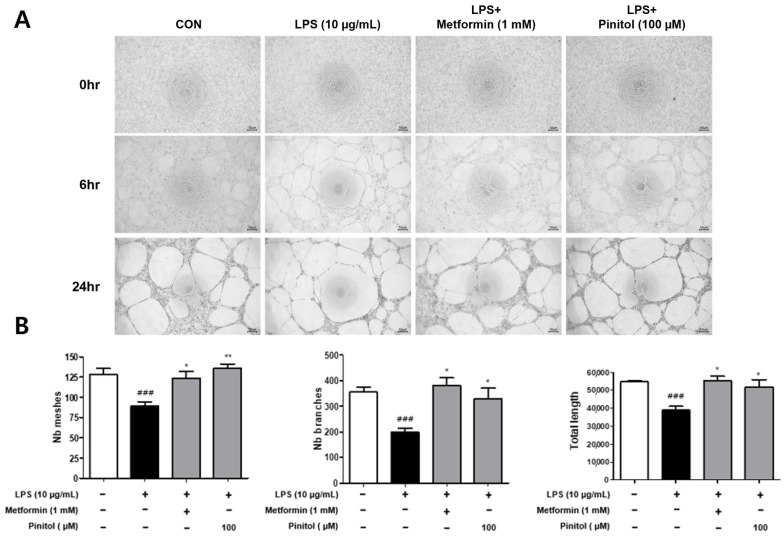
Pinitol recovered the tube formation in LPS-damaged HDMECs. The tube formation assay was conducted on LPS-damaged HDMECs. (**A**) LPS (10 μg/mL) and metformin (1 mM) or pinitol (100 μM) were co-treated. Each image was taken at 6 and 24 h, respectively. Scale bar = 10 µm. (**B**) The total tube length and the number of meshes were quantified using ImageJ software version 2.9.0 (NIH, Bethesda, MD, USA). All data are expressed as mean ± SD (*n* = 3). * *p* < 0.05 compared with the LPS-damaged group. ** *p* < 0.01 compared with the LPS-damaged group. ### *p* < 0.001 compared with the control group.

At the peak of the wound-healing process, the capillary content can be more than three times greater than that of uninjured tissue [55]. The tube formation assay is a rapid and quantitative method for identifying pathways involved in angiogenesis [56,57]. A key feature of this assay is that endothelial cells retain the ability to proliferate and migrate in response to angiogenic signals within 2–6 h [58]. This analytical method can quantify tubes, nodes, loops or meshes, or the tube length [59]. We evaluated the tube formation effects of pinitol in LPS-damaged HDMECs. LPS causes tissue inflammation and decreases endothelial function [60]. As expected, LPS caused a reduction in the number of meshes, branches, and total length of tubes (Figure 7). In contrast, pinitol significantly restored the number of meshes, branches, and total length of tubes in LPS-damaged HDMECs (Figure 7).

### 2.8. Pinitol Improved Specific Cytokine Levels in LPS-Damaged HDMECs

Vascular endothelial growth factor (VEGF) is a potent growth and angiogenic cytokine that plays a crucial role in regulating endothelial cell proliferation and survival [61] while promoting angiogenesis and vascular permeability [62]. Endothelial cell growth and proliferation are essential for angiogenesis [63,64]. Consequently, we performed an ELISA analysis to evaluate the effect of pinitol on VEGF secretion in LPS-damaged HDMECs. LPS causes tissue inflammation and reduces endothelial function. As expected, LPS decreased the expression of VEGF. On the other hand, we observed that pinitol significantly increased VEGF expression in LPS-damaged HDMECs.

To evaluate the effect of pinitol on inflammation, we analyzed IL-6 gene expression in LPS-damaged HDMECs. LPS is known to induce tissue inflammation, impair endothelial function, and increase IL-6 levels [32,44]. LPS treatment increased IL-6 expression levels, whereas pinitol significantly suppressed IL-6 gene expression (Supplementary Figure S1). These data indicate that pinitol may contribute to promoting angiogenesis while modulating the inflammatory response, thereby facilitating the repair and regeneration of diabetic wounds [65,66].

## 3. Discussion

Vascular disease is caused by endothelial dysfunction such as inflammation, injury, and impaired blood flow, with diabetes being one of the leading causes of vascular disease [67,68]. Diabetes can lead to serious complications such as diabetic retinopathy [69], diabetic kidney disease [70], and DFU [71]. Current treatment strategies for managing these diseases primarily rely on medications that regulate blood glucose levels, hypertension, and other related factors [72,73]. However, these drugs can be accompanied by side effects such as muscle pain, indigestion, and liver or kidney damage [74,75,76]. Therefore, there is a need for alternative therapies that can safely improve vascular function.

Metformin has been used as an oral antihyperglycemic agent [77,78]. However, many basic studies have shown that metformin can be a promising candidate for drug repurposing [79]. Recent studies have demonstrated that metformin has direct vascular effects, protecting and improving endothelial function [80,81].

Our study investigated the effects of pinitol as an alternative for improving endothelial function. Pinitol exhibited the ability to enhance cell migration in LPS-damaged HDMECs, highlighting its potential therapeutic role in the proliferation of endothelial cells in wound-healing processes [82]. LPS causes mitochondrial dysfunction, which promotes ROS production [83]. This is a primary cause of endothelial dysfunction [84]. Our results demonstrate that pinitol treatment effectively inhibited ROS and restored mitochondrial function in the LPS-damaged HDMECs (Figure 3 and Figure 4).

**Figure 8:**
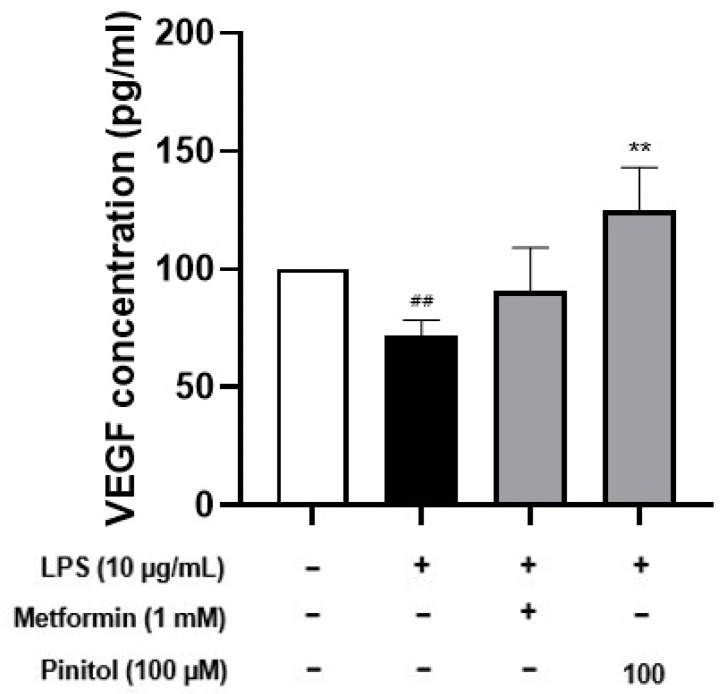
Effects of pinitol on the expression level of VEGF in LPS-damaged HDMECs. The ELISA assay was conducted on LPS-damaged HDMECs. LPS (10 μg/mL) and metformin (1 mM) or pinitol (100 μM) were co-treated for 24 h. VEGF concentrations in the culture supernatants were measured using an ELISA assay. All data are expressed as mean ± SD (*n* = 3). ** *p* < 0.01 compared with the LPS-damaged group. ## *p* < 0.01 compared with the control group.

LPS activated NF-kB through the phosphorylation of IкBα, which translocated to the nucleus and induced the expression of pro-inflammatory cytokines such as IL-1β and IL-6 [85]. The MAPK proteins ERK, JNK, and p38 are important upstream components of NF-kB signaling [86]. Our findings demonstrate that pinitol reduced NF-kB activity and downregulated the MAPK pathway (Figure 5), indicating that pinitol plays a role in modulating the inflammatory response and protecting HDMECs. A few previous reports suggested that pinitol is related to the PI3K/Akt pathway in a rat’s hypothalamus [87] and myocardial apoptosis [88]. Thus, we assumed that pinitol may regulate vascular function through multiple signaling pathways, such as PI3K/Akt, and its synergistic effects with NF-κB and MAPK pathways in HDMECs damaged by external stimuli. Lastly, tube formation is the process by which endothelial cells organize into tubular structures, mimicking the behavior of blood vessels. Our results also show that pinitol repaired damaged tubes in LPS-damaged HDMECs (Figure 7). We further demonstrated that pinitol enhanced the expression level of VEGF, which accelerates tube formation (Figure 8).

Our previous study demonstrated the protective effects of pinitol against UV-induced damage in fibroblasts [31]. UV and LPS trigger cell damage through distinct mechanisms; UV primarily induces oxidative stress and DNA damage, whereas LPS activates the NF-κB and MAPK pathways to promote inflammation through Toll-like receptor 4 [85,86]. This study highlights the potential of pinitol in modulating common signaling pathways involved in these different stress responses, underscoring the need for further mechanistic research.

**Figure 9:**
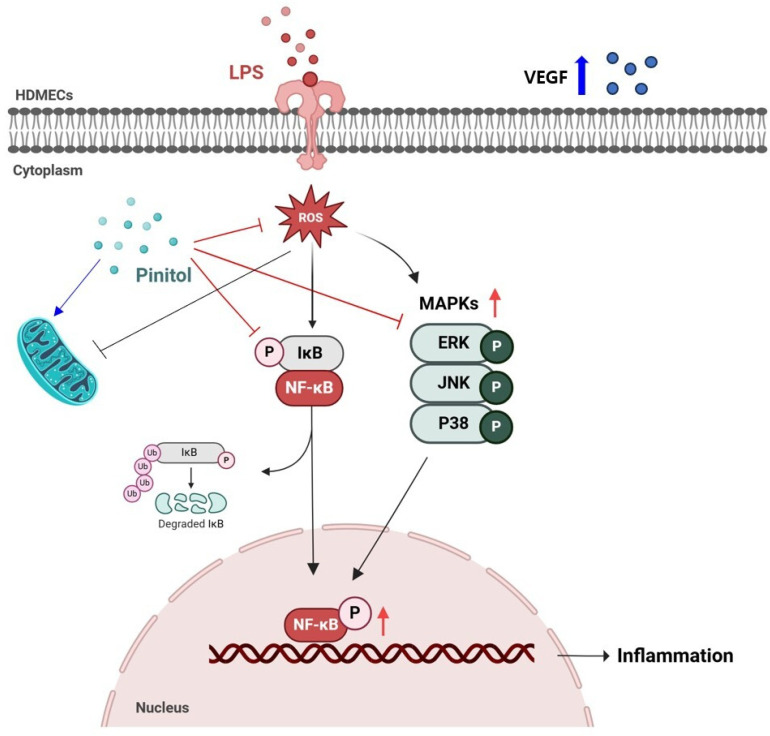
Schematic diagram showing how pinitol inhibited ROS and NF-κB/MAPKs. Pinitol treatment inactivated the NF-κB and MAPK pathways to protect HDMECs from LPS-induced damage, resulting in improved endothelial function. The blue arrows indicated that pinitol had a positive effect directly or indirectly. This scientific illustration was created using BioRender (https://www.biorender.com/library, accessed on 16 December 2024, Toronto, ON, Canada).

This study demonstrated that pinitol alleviated inflammatory responses and improved endothelial function in LPS-damaged HDMECs by downregulating the IκB/NF-κB and MAPK pathways, thereby enhancing skin aging (Figure 9). According to a previous report, pinitol may have potential implications in the prevention and treatment of atherosclerosis [89]. Thus, we suggest that pinitol may be a potential therapeutic agent for endothelial dysfunction even in non-diabetes-related pathologies. This study was conducted based on in vitro experiments, and further preclinical and clinical studies are required to verify the vascular protective effects of pinitol.

## 4. Materials and Methods

### 4.1. Chemicals and Reagents

LPS, metformin, pinitol, and dimethyl sulfoxide were purchased from Sigma Chemical (St. Louis, MO, USA). Dulbecco’s phosphate-buffered saline and 0.25% Trypsin-EDTA were purchased from Welgene Inc. (Gyeongsangbuk-do, Republic of Korea). A 96-well plate, 6-well plate, confocal dish, 100-mm dish, and 75-mm flask were purchased from SPL (Gyeonggi-do, Republic of Korea).

### 4.2. Cell Culture

The HDMECs were obtained from Promo Cell (Heidelberg, Germany) and cultured in an endothelial cell growth medium (Promo Cell, Heidelberg, Germany) with a supplement mix (Promo Cell, Heidelberg, Germany) and 1% penicillin-streptomycin (Welgene, Gyeongsangbuk-do, Republic of Korea). Then, the cells were grown at 37 °C in a 5% CO_2_ incubator. Every 3 days, at 80% or more confluence, cells were harvested using 0.25% Trypsin-EDTA and then transferred to a new 75-mm flask. The medium was supplemented with 30 mM of high glucose in all experiments.

### 4.3. Cell Viability Assay

Cell viability was examined using the EZ-Cytox assay kit (DoGen Bio, Seoul, Republic of Korea). HDMECs were seeded in a 96-well plate at a 2 × 10^4^ cells/well density and incubated for 24 h at 37 °C in a 5% CO_2_ incubator. HDMECs were treated with different concentrations of pinitol (10, 25, 50, 100, and 200 μM) for 24 h. Each well was treated with 10 μL of reagent per 100 μL of endothelial cell suspension. Following 1 h of incubation at 37 °C, the absorbance was recorded at 450 nm using a BioTek Synergy HTX multi-mode reader (BioTek, Winooski, VT, USA).

### 4.4. Wound Healing Assay

HDMECs were seeded in a 6-well plate (SPL, Gyeonggi-do, Republic of Korea) dish at a density of 5 × 10^4^ cells/mL and incubated for 24 h at 37 °C in a 5% CO_2_ incubator. When the cell density reached 80%, a pipette tip (1000 μL) was used to create a scratch on the horizontal line at the bottom of the dish and wash the removed cell debris. The new culture medium was replaced with LPS (10 μg/mL), metformin (1 mM), and pinitol (10, 50, and 100 μM) and incubated for 24 h at 37 °C in a 5% CO_2_ incubator. Images were taken using a Microscope ECLIPSE Ts2 from Nikon (Tokyo, Japan). Wound closure (100%) was analyzed with ImageJ software (NIH, Bethesda, MD, USA).

### 4.5. Tube Formation Assay

The tube formation assay was examined using an angiogenesis assay kit purchased from Cell Biolabs (San Diego, CA, USA). A pre-chilled 96-well plate was coated with 50 μL of Matrigel solution per well and incubated at 37 °C in a 5% CO_2_ incubator for 30 min. The harvested 2.5 × 10^4^ cells/well were resuspended in 150 µL of mediator medium and then seeded onto the ECM gel. After 6 and 24 h, each well was taken with a photograph using a Microscope ECLIPSE Ts2 from Nikon (Tokyo, Japan) and measured using ImageJ software (NIH, Bethesda, MD, USA).

### 4.6. DCF-DA ROS Assay

DCF-DA was examined using a DCFDA-cellular ROS detection assay kit purchased from Abcam (Cambridge, UK). HDMECs were seeded in a confocal dish at a density of 2.5 × 10^4^ cells and incubated for 24 h at 37 °C in a 5% CO_2_ incubator. The cells were stimulated with LPS (10 μg/mL) and treated with metformin (1 mM) or pinitol (100 μM) for 24 h at 37 °C in a CO_2_ incubator. The cells were washed twice with DPBS and then stained with 10 μM 2,7-Dichlorofluoroscein diacetate (DCF-DA) for 15 min. The cells were washed twice with DPBS and DPBS (500 μL) and dispensed for measurement. Fluorescence was measured using an Eclipse Ti2 fluorescence live-cell imaging microscope obtained from Nikon (Tokyo, Japan).

### 4.7. Mitochondrial Membrane Potential Assay

The mitochondrial membrane potential measurement was examined using a mitochondrial membrane potential detection assay kit purchased from Abcam (Cambridge, UK). HDMECs were seeded in a confocal dish (SPL, Gyeonggi-do, Republic of Korea) at a density of 5 × 10^4^ cells and incubated for 24 h at 37 °C in a 5% CO_2_ incubator. Then, the cells were stimulated with LPS (10 μg/mL) and treated with metformin (1 mM) or pinitol (100 μM) for 24 h at 37 °C in a CO_2_ incubator. The cells were washed twice with DPBS and stained with JC-1 staining solution (5 μM) for 15 min. The cells were washed twice with DPBS, and DPBS (500 μL) was dispensed for measurement. Fluorescence was measured using an Eclipse Ti2 fluorescence live-cell imaging microscope from Nikon (Tokyo, Japan).

### 4.8. Immunofluorescence

HDMECs were seeded at a density of 5 × 10^4^ cells/well in a confocal dish (SPL, Gyeonggi-do, Republic of Korea) and incubated for 24 h at 37 °C in a 5% CO_2_ incubator. The cells were co-treated with LPS (10 μg/mL), pinitol (100 μM), and metformin (1 mM) for 24 h. The cells were fixed with 4% paraformaldehyde. After washing 3 times with DPBS, permeability was performed using PBS-T. Then, it was blocked with 3% normal fetal bovine serum for 1 h, and NF-κB and p65 antibody (Cell Signaling Technology, Beverly, MA, USA) was incubated overnight at 4 °C. Anti-rabbit IgG (H + L) cross-adsorbed secondary antibody (Cell Signaling Technology, Beverly, MA, USA) was used. Fluorescence was measured using an Eclipse Ti2 fluorescence live-cell imaging microscope from Nikon (Tokyo, Japan).

### 4.9. Western Blot Analysis

HDMECs were seeded at a density of 5 × 10^4^ cells/well in a 100-mm dish (SPL, Gyeonggi-do, Republic of Korea) and incubated for 24 h at 37 °C in a 5% CO_2_ incubator. The cells were co-treated with LPS (10 μg/mL), pinitol (10, 50, and 100 μM), and metformin (1 mM) for 24 h. The cells were washed twice with DPBS (Welgene, Gyeongsangbuk-do, Korea) and lysed using RIPA buffer (Thermo Fisher Scientific, Waltham, MA, USA). Then, the cells were centrifuged at 15,000 rpm at 4 °C for 10 min. The protein concentration was measured using a Pierce™ BCA protein assay kit (Thermo Fisher Scientific, Waltham, MA, USA) according to the manufacturer’s instructions. The 30 µg of proteins was separated on 10% sodium dodecyl sulfate-polyacrylamide gel electrophoresis and then transferred onto a PVDF membrane (Cytiva, Marlborough, MA, USA). The membranes were blocked with 5% non-fat skim milk (Bio-Rad, Hercules, CA, USA) in TBS-T (0.1% Tween-20) for 2 h at room temperature and incubated overnight with primary antibodies (p-ERK, p-JNK, p-p38, p-IκBα, p-NFκB, ERK, JNK, p38, IκBα, NFκB, and actin) (Cell Signaling Technology, Beverly, MA, USA) at 4 °C. The membranes were washed three times at room temperature with TBS-T. They were incubated with HRP-conjugated secondary antibody for 2 h at room temperature and washed with TBS-T three times. The immunoreactive bands were detected using the ECL reagent (Cytiva, Marlborough, MA, USA), and images were visualized with an Invitrogen iBright 1500 (Waltham, MA, USA). The results were analyzed using ImageJ software (NIH, Bethesda, MD, USA).

### 4.10. ELISA Assay

The ELISA assay was examined using a Human VEGF-A ELISA Kit (Thermo Fisher Scientific, Waltham, MA, USA). HDMECs were seeded in a 96-well plate dish at a density of 2.5 × 10^4^ cells/well. The cells were stimulated with LPS (10 μg/mL) and treated with metformin (1 mM) or pinitol (100 μM) for 24 h. Then, 50 µL of the cell media supernatant from the cultured plate and 50 µL of sample diluent were added. The plate was covered with an adhesive film and incubated at room temperature for 2 h. The microwell strips were washed 6 times using a wash buffer, and 100 µL of biotin-conjugate was added. The plate was covered with an adhesive film and incubated at room temperature for 1 h. The microwell strips were washed 6 times using a wash buffer, and 100 µL of diluted streptavidin-HRP was added to all wells, including the blank wells. The plate was covered with an adhesive film and incubated at room temperature for 1 h. The microwell strips were washed 6 times using a wash buffer, and 100 µL of TMB substrate solution was added to all wells and incubated at room temperature for about 30 min. Then, 100 µL of stop solution was added to each well. The absorbance was recorded at 450 nm using a Bio Tek Synergy HTX multi-mode reader (Winooski, VT, USA).

### 4.11. Quantitative Real-Time Polymerase Chain Reaction

HDMECs were seeded in a 6-well plate at a density of 10.0 × 10⁴ cells per well and cultured for 24 h. Subsequently, the cells were treated with 10 μg/mL LPS, 1 mM metformin, and 100 μM pinitol for 24 h. The cells were washed twice with DPBS. The total RNA was isolated using TRIzol reagent (Thermo Fisher Scientific, Waltham, MA, USA), and 2 μg of RNA was reverse transcribed into cDNA using a RevertAid First Strand cDNA Synthesis Kit (Thermo Fisher Scientific, Waltham, MA, USA). A quantitative real-time polymerase chain reaction (qRT-PCR) was performed using the TaqMan Universal Master Mix II with UNG. The reaction mixture consisted of 6 μL DEPC-treated water, 10 μL TaqMan Universal Master Fast Mix II, 3 μL cDNA, and 1 μL of each primer.

### 4.12. Statistical Analysis

All results are expressed as the mean ± standard deviation (SD) of three independent experiments. According to Tukey’s Multiple Comparison Test, all statistical analyses were performed using GraphPad Prism 5.0 software (San Diego, CA, USA) through a one-way analysis of variance (ANOVA).

## Data Availability

The data from this study are available upon demand from the corresponding author.

## Supplementary Materials

The following supporting information can be downloaded at https://www.mdpi.com/article/10.3390/molecules30071513/s1. Figure S1: Effects of pinitol on the expression level of IL-6 in LPS-damaged HDMECs.
