# Supplementary material for: Pinitol Improves Lipopolysaccharide-Induced Cellular Damage in Human Dermal Microvascular Endothelial Cells
Source: Molecules. 2025 Mar 28;30(7):1513. doi: 10.3390/molecules30071513 (PMC11990420; doi:10.3390/molecules30071513)
Supplement: Supplementary file 1 [file molecules-30-01513-s001.zip › molecules-3343880-supplementary.pdf]

Supplementary Figure S1.

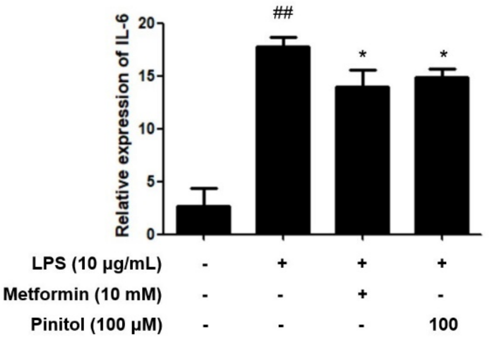

Figure S1. Effects of pinitol on the expression level of IL-6 in LPS-damaged HDMECs.

The real-time quantitative RT-PCR analysis was conducted on LPS-damaged HDMECs. LPS (10 μg/mL) and metformin (1 mM) or pinitol (100 μM) were co-treated for 24 hours. The expression level of IL-6 was measured. All data were expressed as mean ± SD (n=3). *p < 0.05 compared with the LPS-damaged group. ##p < 0.01 compared with the control group.
